# A longitudinal cohort study of HIV ‘treatment as prevention’ in gay, bisexual and other men who have sex with men: the Treatment with Antiretrovirals and their Impact on Positive And Negative men (TAIPAN) study protocol

**DOI:** 10.1186/s12879-016-2073-2

**Published:** 2016-12-12

**Authors:** D. Callander, M. Stoové, A. Carr, J. F. Hoy, K. Petoumenos, M. Hellard, J. Elliot, D. J. Templeton, S. Liaw, D. P. Wilson, A. Grulich, D. A. Cooper, A. Pedrana, B. Donovan, J. McMahon, G. Prestage, M. Holt, C. K. Fairley, N. McKellar-Stewart, S. Ruth, J. Asselin, P. Keen, C. Cooper, B. Allan, J. M. Kaldor, R. Guy

**Affiliations:** 1 The Kirby Institute, UNSW Australia, Wallace Wurth Building, Sydney, NSW 2052 Australia; 2 Burnet Institute, Melbourne, VIC Australia; 3 St Vincent’s Hospital, Sydney, NSW Australia; 4 Monash University, Melbourne, VIC Australia; 5 Alfred Hospital, Melbourne, VIC Australia; 6 RPA Sexual Health, Community Health, Sydney Local Health District, Sydney, NSW Australia; 7 Central Clinical School, The University of Sydney, Sydney, NSW Australia; 8 School of Public Health and Community Medicine, UNSW Australia, Sydney, NSW Australia; 9 Sydney Sexual Health Centre, Sydney Hospital, Sydney, NSW Australia; 10 Australian Research Centre in Sex, Health and Society, La Trobe University, Melbourne, VIC Australia; 11 Centre for Social Research in Health, UNSW Australia, Sydney, NSW Australia; 12 Melbourne Sexual Health Centre, Melbourne, VIC Australia; 13 ACON Northern Rivers, Lismore, NSW Australia; 14 Victorian AIDS Council, Melbourne, VIC Australia; 15 PositiveLife New South Wales, Sydney, NSW Australia; 16 Living Positive Victoria, Melbourne, VIC Australia

**Keywords:** HIV, Treatment as prevention, Cohort, Gay men

## Abstract

**Background:**

Australia has increased coverage of antiretroviral treatment (ART) over the past decade, reaching 73% uptake in 2014. While ART reduces AIDS-related deaths, accumulating evidence suggests that it could also bolster prevention efforts by reducing the risk of HIV transmission (‘treatment as prevention’). While promising, evidence of community-level impact of treatment as prevention on reducing HIV incidence among gay and bisexual men is limited. We describe a study protocol that aims to determine if scale up of testing and treatment for HIV leads to a reduction in community viraemia and, in turn, if this reduction is temporally associated with a reduction in HIV incidence among gay and bisexual men in Australia’s two most populous states.

**Methods:**

Over the period 2009 to 2017, we will establish two cohorts making use of clinical and laboratory data electronically extracted retrospectively and prospectively from 73 health services and laboratories in the states of New South Wales and Victoria. The ‘positive cohort’ will consist of approximately 13,000 gay and bisexual men (>90% of all people living with HIV). The ‘negative cohort’ will consist of at least 40,000 HIV-negative gay and bisexual men (approximately half of the total population). Within the negative cohort we will use standard repeat-testing methods to calculate annual HIV incidence. Community prevalence of viraemia will be defined as the proportion of men with a viral load ≥200RNA copies/mm^3^, which will combine viral load data from the positive cohort and viraemia estimates among those with an undiagnosed HIV infection. Using regression analyses and adjusting for behavioural and demographic factors associated with infection, we will assess the temporal association between the community prevalence of viraemia and the incidence of HIV infection. Further analyses will make use of these cohorts to assess incidence and predictors of treatment initiation, repeat HIV testing, and viral suppression.

**Discussion:**

This study will provide important information on whether ‘treatment as prevention’ is associated with a reduction in HIV incidence at a community level among gay and bisexual men.

## Background

Since 1996, combination antiretroviral therapy (ART) for HIV infection has been used to treat HIV by reducing the progression to AIDS and preventing AIDS-related deaths. In recent years, ART has been acknowledged as a key biomedical component in reducing onward HIV transmission. Known as ‘treatment as prevention’, the potential for this public health approach to control HIV transmission was evident in the landmark HIV Prevention Trials Network (052) randomised control trial, which in 2011 observed a 96% reduction in HIV transmission to heterosexual partners of HIV-positive individuals allocated to early ART compared with those with delayed therapy [1]. More recently, observational cohorts of both same and opposite sex couples have provided further supporting evidence, with no linked HIV transmissions among those with sustained viral suppression despite thousands of acts of condomless sex [2, 3]. These findings have formed the basis of international recommendations to employ treatment as prevention as an additional strategy to eliminate HIV [4].

For treatment as prevention to have greatest impact, HIV testing and treatment coverage must be high and treatment must be initiated soon after an infection is diagnosed [5]. National estimates from 2014 showed that 73% of people diagnosed with HIV in Australia were on ART [6] and observational research suggests that treatment coverage has increased over time [7]. Such increases in treatment coverage likely reflect changes to HIV treatment guidelines. While earlier guidelines only recommended ART initiation for people with CD4 cell count below 350 cells/mm^3^ in 2012 they were updated to include consideration for those with counts below 500 cells/mm^3^ and most recently recommended treatment initiation as soon as possible after diagnosis independent of CD4 cell count [8]. Additionally, a number of policy and structural barriers to HIV treatment were addressed, including the removal of CD4 cell count criteria for medication reimbursement through the national health scheme [9] and in the state of New South Wales the removal of upfront co-payments associated with treatment dispensing [10].

Alongside treatment, efforts have been made to increase HIV testing in Australia through a number large-scale health promotion campaigns and new initiatives to improve access to testing services. Community surveys of gay and bisexual men in 2014 found that 90% had ever received an HIV test with two-thirds tested within the previous year, proportions that have been generally stable over the past ten years [11]. Among men at highest risk of infection, Australian guidelines recommend at least six-monthly HIV testing [12] but clinical data show only about half of higher-risk men in 2014 returned for a follow-up test within six months (up from 40% in 2009) [13]. Thus, there have been some improvements in HIV testing among gay and bisexual men but to a lesser degree than for treatment.

Despite improvements in HIV treatment and testing coverage, annual HIV notifications in Australia increased by 22% between 2004 and 2012 [6]. In response, many Australian jurisdictions moved to expand ‘treatment as prevention’ initiatives and in 2014 all jurisdictional health ministers committed to a target of virtual HIV elimination by 2020. In recent years, there has been an increased focus on testing and treatment initiatives, implemented through partnerships between governments, clinicians, community organisations and researchers. These changes represent a major effort nationally to advance treatment as prevention.

This paper describes a study titled ‘TAIPAN’ (Treatment with Antiretrovirals and their Impact on Positive And Negative Men), which aims to establish two large longitudinal cohorts between 2009 and 2017 to evaluate if the scale up HIV testing and treatment leads to a reduction in community viraemia and if this reduction is temporally associated with a reduction in HIV incidence among gay and bisexual men.

## Methods

### Study design

This study will involve a longitudinal cohort design using de-identified, electronic medical records extracted for the period 1 January 2009 to 31 December 2017.

### Aims

The primary aim of the study is to determine the temporal association between the community prevalence of HIV viraemia and the incidence of HIV infection among gay and bisexual men at a community-level. The secondary aims are: i) to assess the association between changes in guidelines and policies and earlier uptake of HIV treatment among gay and bisexual men, ii) to identify incidence and predictors of repeat HIV testing among HIV negative gay and bisexual men, and iii) to identify incidence and predictors of supressed HIV viral load among HIV positive gay and bisexual men.

### Setting

Two study cohorts will be established in the two Australian states where approximately 65% of gay and bisexual men in Australia live: New South Wales (population ~7.6 million) and Victoria (population ~5.9 million).

### Cohorts

Study cohorts will be established using de-identified data extracted from electronic patient medical records. These data will be collected via an existing health sentinel surveillance network of clinics and laboratories known as the *Australian Collaboration for Coordinated Enhanced Sentinel Surveillance of Sexually Transmissible Infections and Blood Borne Viruses* (ACCESS). The existing network will be expanded from 50 health services and pathology laboratories in New South Wales and Victorian to a total of 73, including: 39 publicly-funded sexual health clinics, 10 general practice clinics with medium to high caseloads of gay and bisexual men (50 or more patients per year), six hospital HIV outpatient clinics, four community-led HIV testing services, and 14 private and public pathology laboratories.

Expansion of the surveillance network will aim to capture 95% or more of all HIV viral load tests conducted in both states as well as 100% of HIV diagnoses. These targets will be achieved by recruiting all laboratories that conduct HIV viral load testing as well all reference laboratories responsible for confirmatory HIV Western Blot testing. Table 1 outlines the service types and criteria used to identify health services and laboratories relevant to TAIPAN.

**Table 1:** Health service and pathology laboratory types and criteria for the TAIPAN project (*n* = 73)

	Health services	Pathology laboratories
Service types	Sexual health clinics (*n* = 39) General practice clinics (*n* = 10) Hospitals (*n* = 6) Community-led health services (*n* = 4)	Publicly-funded (*n* = 6) Private (*n* = 8)
Criteria (any)	≥50 HIV gay/bisexual men annually ≥20 HIV positive male patients annually ≥5 HIV diagnoses annually	Any confirmatory HIV testing Any HIV viral load testing

### Participants

Using de-identified data extracted from ACCESS sites, two patient cohorts of gay and bisexual men will be established: one comprising HIV positive men (‘positive cohort’) and the other HIV negative men (‘negative cohort’). The positive cohort will consist of approximately 13,000 HIV positive gay and bisexual men (approximately 90% of all gay and bisexual men with HIV in both states) and the negative cohort will consist of at least 40,000 HIV negative gay and bisexual men (approximately half of the population).

Both cohorts will be limited to men aged 16 years and older for whom there is at least once record indicating a history of sexual contact with other men. Indicators of same sex contact can be behavioural (i.e., self-reporting same sex partners), identity-based (i.e., recorded sexual orientation as gay or bisexual), or procedural (i.e., collection of an anal swab for STI testing). The positive cohort will include patients recorded as HIV positive or those who receive an HIV diagnosis during the study period. The negative cohort will include patients whose first record of HIV testing during the study period is negative. If during the course of the study a person is diagnosed with HIV, they will be reclassified to the positive cohort. Follow-up during the study period will be based on records from the participating services as well as the pathology conducted by participating laboratories.

### Study outcomes

Table 2 outlines the study aims, relevant cohorts, and study outcomes.

**Table 2:** Overview of TAIPAN research aims, study cohorts involved and outcome indicators

	Cohort(s)	Outcome indicators
Primary aim		
Determine the relationship between viraemia and incidence of new HIV infections among gay and bisexual men	Negative Positive	• HIV incidence • Viraemia prevalence
Secondary aims		
1. Assess the association between changes in guidelines and policies and earlier uptake of HIV treatment among gay and bisexual men	Positive	• Incidence of treatment initiation
2. Identify incidence and predictors of repeat HIV testing among HIV negative gay and bisexual men	Negative	• Incidence of repeat testing (second HIV test within a 12 month period)
3. Identify incidence and predictors of supressed HIV viral load among HIV positive gay and bisexual men	Positive	• Incidence of viral suppression

#### Outcome 1

The community prevalence of viraemia is defined as the proportion of gay and bisexual men (diagnosed and undiagnosed) with a viral load of ≥200 RNA copies/mm^3^. A viral load of ≥200 was selected as this is a commonly-used clinical marker of viral suppression and a recent meta-analysis found no transmission of HIV among individuals with less than 200 RNA copies/mm^3^ [14]. Community prevalence of viraemia was selected as it has been demonstrated to have the strongest correlation with HIV incidence over other measures (e.g., mean viral, treatment coverage) [15]. Our measure of viraemia will comprise the following four components:

1. Annual prevalence of viraemia: will be calculated within the positive cohort using the annual prevalence of viral load test results of ≥200 RNA copies/mm^3^ (‘viraemia’) at each patients last viral load test within a calendar year. We will conduct sensitivity analyses to include any viral load test ≥200 RNA copies/mm^3^ in a year and also using a higher threshold of ≥1000 RNA copies/mm^3^.
2. Annual prevalence of diagnosed HIV infection among gay and bisexual men: will be estimated using population prevalence techniques developed by and used for Australia’s national HIV surveillance [6]. This approach produces a prevalence estimate using data on HIV diagnoses from Australia’s National HIV Registry, adjusting for duplicate entries, deaths, and migration outside of Australia. For this estimate we will focus on HIV diagnoses where homosexual contact is identified as the route of transmission.
3. Annual prevalence of undiagnosed HIV infection among gay and bisexual men: will be estimated using standard back-project models [16] and validated against a separate bio-behavioural survey conducted in 2013–2014 and to be repeated in 2017 [17].
4. Prevalence of viraemia in gay and bisexual men with undiagnosed HIV infection: will be calculated using an estimate of undiagnosed infection obtained from the back-projection models coupled with the assumption that all undiagnosed men have viral loads of ≥200 RNA copies/mm^3^. We will also consider different viral load levels among undiagnosed men by using data from the National HIV Registry and stratifying viral load at diagnosis by HIV testing history (i.e., reported time since HIV test prior to diagnosis).

**Fig. 1:**
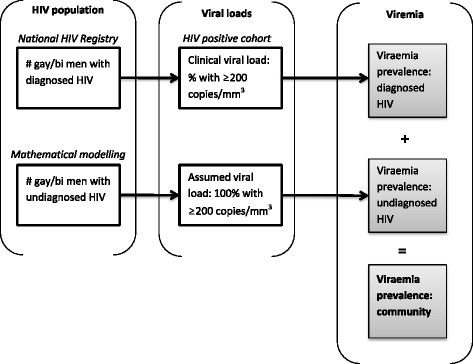
Method for calculating the prevalence of HIV viraemia among diagnosed and undiagnosed gay and bisexual men

The combination of these components to generate community viral load is depicted in Fig. 1. We will calculate an annual community prevalence of viraemia in gay and bisexual men (diagnosed and undiagnosed) by combining (i) the annual prevalence of viraemia among those who have been diagnosed with the estimates of HIV prevalence, and (ii) the annual prevalence of viraemia among undiagnosed men with the estimates of undiagnosed infections.

#### Outcome 2

HIV incidence will be calculated using repeat testing among the negative cohort, [18] which will consider patients with two or more HIV tests for whom the first test was negative. We expect that more than 80% of gay and bisexual men will have at least two tests over the study period. An incident infection will be defined as an HIV diagnosis following a negative test and time at risk will be calculated as the time between each patient’s first and last test or a patient’s first test and HIV diagnosis. The number of incident infections will be divided by the person time (in years) at risk.

#### Outcome 3

Data from the positive cohort will also be used to calculate the incidence of ART initiation (i.e., the point at which ART is initiated) with the follow-up period defined as the time after HIV diagnosis (person years of infection). This variable will be restricted only to men diagnosed with HIV during the study period.

#### Outcome 4

Data from the negative cohort will be used to calculate the incidence of repeat diagnostic HIV testing. The incident event is defined as the point at which a patient achieves a second HIV test within six weeks to 12 months of a previous test, with follow-up time defined as the period between a patient’s first HIV test and last clinical encounter. Follow-up time will cease if a patient has no recorded service event of any kind within a two year period but will resume at the point of future service events (i.e., reengages with a participating service). Follow-up will also cease if a patient is diagnosed with HIV.

#### Outcome 5

Among the positive cohort, the incidence of viral suppression will be calculated. Viral suppression is defined as two consecutive viral load tests of <200 RNA copies/mm^3^ separated by at least three months; the first test will be taken as the point of suppression for calculating time. This variable will only be calculated among patients with no history of previous treatment and will exclude those for whom there is no or only one viral load test result. We will conduct a sensitivity analysis with a higher viral load threshold of <1000 RNA copies/mm^3^.

### Covariates

Covariates considered for this study’s analyses will include patient demographic variables (area of residence, age, indigenous status, country of birth, if Australia’s public health insurance scheme ‘Medicare’ was recorded), clinical information (HIV status, STI diagnoses, CD4 cell count and viral load results, hepatitis B and C co-infection), behavioural information (gender(s) of sexual partner, condom use, injecting drug use, recent sex work, and sexual partner numbers) and service provision - related variables (clinic location - urban vs regional/remote, service type - general practice, sexual health clinic, hospital, community-led service). Additionally, treatment information and treatment type will be used to assess the use of HIV antiretrovirals among the negative cohort as pre- and/or post-exposure prophylaxis (‘PrEP’ and ‘PEP’). The only variables likely to be missing from a substantial number of records relate to sexual risk behaviour. For this information we will draw on records from multiple sources and use a recorded diagnosis of rectal chlamydia and gonorrhoea as a surrogate marker of risk [19]. Not all covariates will be relevant to every analysis.

### Data sources

Routine and de-identified patient data will be extracted from participating service databases, encrypted, and transmitted electronically to a secure server using customised software known as GRHANITE™ [20]. These extractions will provide the data relevant to the co-variate and study outcomes described above. The accuracy of GRHANITE™ has been assessed via internal reliability checks, which showed that the software correctly classified all pathology results as positive or negative, and by comparing extracts with external laboratory data, which found 92–95% concordance [21].

Data quality checks will be conducted biannually and will include a review of testing numbers to identify monthly totals exceeding one standard deviation from the mean. We will also compare data between service types (i.e., laboratory tests recorded at participating health services compared to tests reported by the laboratories relevant to that service) and by comparing the number of HIV and STI diagnoses recorded in patient medical record systems to those reported to the jurisdictional health departments. We will also work with participating sites to compare aggregate outputs produced by their systems internally with those extracted via GRHANITE™.

The GRHANITE™ extraction software de-identifies patient data by performing one-way cryptographic transformation of patient details using a secure hash algorithm. The resultant ‘hashes’ are, therefore, based on patient details but completely anonymous; they are generated before data are transmitted from a health service or laboratory to ensure that potentially identifying details are not extracted from a participating service [21]. Patient hashes are generated using four complex algorithms that combine identifiable patient details (e.g., given and surnames, date of birth) to generate unidentifiable strings of code. Using a combination of one or more of these codes, patients will be linked probabilistically between and within health services and laboratories. Matching patients on four out of four hashes represents the highest quality match or what might be considered near certainty while matching on fewer hashes suggests less certainty. All matches will be assessed by comparing available demographic data between services (e.g., widely disparate ages for one patient between multiple services).

### Data analyses

TAIPAN will undertake four separate analyses to address the study aims (Table 2).

TAIPAN’s primary aim will be addressed by examining the temporal relationship between community prevalence of viraemia and incidence of HIV infection in gay and bisexual men. For each year of follow up, the community prevalence of HIV viraemia as determined by the calendar year will be entered as an independent variable in the regression. In addition to estimating the effect of community prevalence of viraemia, we will control at the individual level for other demographic, clinical and behavioural determinants of acquisition of new HIV infection, and recent sexual risk practices. We will use random effects Poisson models to undertake multi-level analyses that will allow us to include group-related variables (community prevalence of viraemia), individual variables (demographic, clinical, and behavioural) and time.

The study period will be divided into three-year periods defined by treatment guidelines and policy. The first period will encompass 2009–2011 and serve as a ‘before’ treatment as prevention period. The second period will encompass 2012–2014 and represent a transition period given that several Australian jurisdictions began to expand treatment as prevention initiatives during this time. And the third period will encompass 2015–2017, representing the full implementation of treatment as prevention given that early ART initiation was recommended for both individual and public health purposes. In regards to time lags, we assume that HIV incidence is affected by the estimated prevalence of virological suppression from the preceding year.

The second analysis will examine the temporal relationship between the three time periods described above and the incidence of treatment initiation among men newly diagnosed with HIV (Secondary Aim 1). Using the study periods described above in a regression analysis, incidence of treatment initiation among men newly diagnosed with HIV will be the outcome variable while controlling for clinical, demographic and behavioural factors. Given its historical role in determining when to initiate treatment, CD4 cell count at diagnosis and HIV viral load will also be included in the multivariate model.

The third analysis will determine incidence and predictors of repeat HIV testing (Secondary Aim 2). To calculate annual estimates we will allocate follow-up time and the incident of repeat testing (i.e., the point at which a second test is achieved) to the calendar year in which it occurs. With this approach it is possible that a patient might achieve multiple incidents of repeat testing in one calendar year. In addition to evaluating changes in the incidence of repeat HIV testing over time, this analysis will employ a regression analysis to identify demographic, behavioural and clinical factors associated with repeat testing incidence among gay and bisexual men in the negative cohort.

The fourth analysis will estimate the incidence of viral suppression among those newly diagnosed and predictors of incidence of viral suppression among the positive cohort (Secondary Aim 3). This analysis will employ Cox proportional hazards regression. Further, we will estimate the median time from diagnosis to first viral suppression by calendar year among those who are diagnosed during the study period, stratified by CD4 cell count at diagnosis. For this analysis and all others, statistical significance will be set at *p* < 0.05.

### Sample size

Sample size calculations are based on the estimated number of gay and bisexual men diagnosed with HIV annually in New South Wales and Victoria, [6] and assuming a modelling-derived 30% reduction in infections to 420 per year in 2015–2017 (i.e., following the full implementation of treatment as prevention) [22]. For HIV incidence (Primary Aim) an estimated 80% of men diagnosed annually will have some HIV testing history, [23, 24] which equates to 336 incident cases annually and will provide 80% power to detect annual declines in the incidence rate of at least 20% (hazard ratio [HR] = 0.80). Regarding treatment initiation (Secondary Aim 1), assuming a rate of treatment initiation of 36 per 100 person years (unpublished data from Australian HIV Observational Database) and an estimated 420 men newly diagnosed per year, our analysis will have 80% power to detect annual relatives changes in treatment initiation after a new HIV diagnosis of at least 36% (HR = 1.36).

For repeat HIV testing (Secondary Aim 2), it is estimated that 80% of men in the negative cohort will be tested at least once annually for HIV [23, 24] and that 25% will have one further test in the year [25]. If the cohort contains approximately 40,000 men then this translates into 8000 repeat testers annually, which will provide sufficient power to detect at least a 6% annual increase in the incidence of repeat testing (HR = 1.06). And for viral suppression (Secondary Aim 3), if we assume that 90% of newly diagnosed men will start treatment [26] then we will have 80% power to detect a 15% increase in the annual incidence of viral suppression (HR = 1.15). Trend tests over multiple years will have greater power as will combining over multiple years as per the three-year study periods relative to the implementation of treatment as prevention (see Data Analyses).

## Discussion

Although treatment as prevention has already been shown to be highly effective among serodiscordant couples, further evidence is required to determine its community-level impact on HIV incidence among gay and bisexual men. This issue is particularly relevant in the Australian context, where testing and treatment coverage is high but where there has been no decline in annual HIV diagnoses [6]. The two large cohorts established in this study will provide detailed information on the level of testing and treatment coverage that can be achieved and, in the turn, the impact on HIV incidence.

While clinical trial results and modelling studies have fostered optimism about the treatment as prevention’s public health potential, the ultimate reduction of incidence may be somewhat less than expected in gay and bisexual men [26]. In Australia, an estimated 12% of people living with HIV are undiagnosed [6] with the average time between infection and diagnosis estimated to be between two and three years [22]. Undiagnosed infections contribute disproportionately to HIV transmission due to higher viral loads and sexual practices [5]. It is, therefore, a key strength of this study that it will consider the roles of diagnosed and undiagnosed HIV in onward transmission among gay and bisexual men.

There have been only a few community-level studies of treatment as prevention in gay and bisexual men [27–30]. A consistent limitation in all these studies is the indirect measurement of HIV incidence, calculated either via mathematical modelling or derived from case notifications. Although case notifications in a highly tested population may be close to reflecting incidence, in Australia nearly a quarter of diagnoses are considered ‘late’ (CD4 cell counts of <350/mm^3^) [6] and trends in notifications can be influenced by fluctuations in diagnostic testing. One large, community-level study of heterosexuals in Africa measured HIV incidence directly between 2009–2012 and demonstrated a strong correlation between treatment coverage and HIV incidence at a community-level [31]. These findings, however, cannot be applied to gay and bisexual men due to population differences in sexual behaviour and HIV incidence. In addition, the levels of HIV testing and treatment coverage reported in this study were of the levels achieved in Australia more than a decade ago. Furthermore, any community-level evaluation of treatment as prevention must account for potential confounders, including other prevention and risk reduction strategies and notably the uptake of PrEP.

Keys strengths of the TAIPAN study design are its ability to directly measure HIV incidence among gay and bisexual men, the inclusion of undiagnosed men in assessing community prevalence of viraemia, and the large patient cohorts. There are, however, some limitations to consider. First, the HIV negative cohort will include approximately half of all gay and bisexual men across both states. Although very large, the cohorts are unlikely to be fully representative of all gay and bisexual men in New South Wales and Victoria. We believe it is reasonable to assume, however, that any temporal relationships and predictors of study outcomes we detect in a cohort of this size will be applicable to the general population of gay and bisexual men. Second, during 2016 and 2017 access to PrEP has and will be expanded in both New South Wales and Victoria. This parallel prevention initiative may impact on HIV incidence but we will be able to assess and control for PrEP uptake in the multivariate analyses through prescribing data in the HIV negative cohort. Third, optimism associated with these new prevention strategies may foster changes in men’s sexual behaviour and potentially undermine the efficacy of treatment as prevention. As TAIPAN is not able to collect detailed sexual risk behaviour data, this question will be explored via separate studies.

The TAIPAN study will contribute to the growing body of literature that seeks to understand the impact of HIV treatment as prevention in a range of settings and population groups. These study outcomes will have implications for policy and practice in resource-rich countries where the epidemic mainly affects gay and bisexual men. Considering there have been significant investments in HIV testing and treatment initiatives in recent years in Australia and overseas, it is vital to assess the impact of treatment as prevention on HIV incidence.

## Abbreviations

ACCESS: Australian Collaboration for Coordinated Enhance Sentinel Surveillance of Sexually Transmissible Infections and Blood Borne Viruses
AIDS: Acquired immune deficiency syndrome
ART: Antiretroviral therapy
HIV: Human immunodeficiency virus
PEP: Post-exposure prophylaxis
PrEP: Pre-exposure prophylaxis
TAIPAN: Treatment with antiretrovirals and their impact on positive and negative men
